# A New Rapid Methodological Strategy to Assess BRCA Mutational Status

**DOI:** 10.1007/s12033-012-9646-0

**Published:** 2013-01-26

**Authors:** Emilia Vuttariello, Marco Borra, Celeste Calise, Elvira Mauriello, Stefano Greggi, Aldo Vecchione, Elio Biffali, Gennaro Chiappetta

**Affiliations:** 1 Functional Genomic Unit, National Cancer Institute, Fondazione “G.Pascale”, Via Mariano Semmola, 80131 Naples, Italy; 2 Molecular Biology Service, Stazione Zoologica “A. Dohrn”, Villa Comunale, 80121 Naples, Italy; 3 Gynecologic Oncology Unit, Cancer Institute, Fondazione “G.Pascale”, Via Mariano Semmola, 80131 Naples, Italy; 4 National Cancer Institute, Fondazione “G.Pascale”, Via Mariano Semmola, 80131 Naples, Italy

**Keywords:** BRCA1, BRCA2, Hereditary breast and ovarian cancer (HBOC), Genetic testing, Direct sequencing

## Abstract

Hereditary cancers account for approximately 10 % of breast and ovarian cancers. Mutations of the BRCA1 and BRCA2 genes, encoding two proteins involved in DNA repair, underlie most cases of such hereditary cancers. Women with BRCA mutations develop breast cancer in 50–80 % of cases and ovarian cancer in 10–40 % of cases. Assessing BRCA mutational status is needed to direct the clinical management of women with predisposition to these hereditary cancers. However, BRCA screening constitutes a bottleneck in terms of costs and time to deliver results. We developed a PCR-based assay using 73 primer pairs covering the entire coding regions of BRCA1 and BRCA2. PCR primers, containing at the 5’ end the universal M13 primer sequences, were pre-spotted in 96-well plates. Following PCR, direct sequencing was performed using M13 primers, allowing to standardize the conditions. PCR amplification and sequencing were successful for each amplicon. We tested and validated the assay on 10 known gDNAs from patients with Hereditary breast and ovarian cancer (HBOC). Our strategy is a promising time and cost-effective method to detect BRCA mutations in the clinical setting, which is essential to formulate a personalized therapy for patients with HBOC.

## Introduction

In this study we present a methodology for the direct sequencing of *BRCA* genes through a simple workflow implementable in a diagnostic lab.

Breast and ovarian cancer are the leading cause of cancer death in women worldwide. Most tumors are considered sporadic, whereas the remaining 5–10 % is inherited as autosomal dominant disease and defined as Hereditary breast and ovarian cancer (HBOC) [1].

*BRCA1* and *BRCA2* were identified in the early 90s as the genes that confer a higher risk of developing this hereditary cancer syndrome [2, 3].

Patients with HBOC, differently from those with the sporadic type of cancer, are characterized by a young age of onset and the presence in the family of numerous cases of cancer, not only of breast cancer but also ovarian and/or cancer affecting other organs. Furthermore, although rarely, even males can develop breast cancer in these families [4].

Women bearing an alteration in *BRCA1* or *BRCA2* develop during their lifespan a breast cancer in 50–80 % of cases and an ovarian cancer in 20–40 % of cases (carriers of *BRCA1* mutation) or in 10–20 % of cases (carriers of *BRCA2* mutation) [5].

*BRCA1* and *BRCA2* are tumor suppressor genes, with autosomal dominant transmission and high penetrance. Specific *BRCA* mutations can confer a different risk of disease, consistent to the fact that breast and ovarian cancer are multifactorial diseases, which can be influenced by many environmental and/or genetic factors affecting *BRCA1* or *BRCA2* penetrance [6].

*BRCA1* and *BRCA2* are the two main breast cancer susceptibility genes for which mutation recognition is important to assess cancer risk and to identify more suitable treatment strategies [7].

*BRCA1* is located on chromosome 17. It consists of 24 exons (with exon 11 constituting 61 % of the coding region), which are distributed over a region of approximately 100 kb, and encodes a protein of 1,863 amino acids. *BRCA1* is regulated by two separate promoters inducing the transcription of two mRNAs with different 5′UTRs. In some cancers BRCA1 downregulation occurs following the switch from the expression of 5′UTRa, which enables an efficient protein translation, to the expression of 5′UTRb, which, conversely, strongly inhibits translation [8].

*BRCA2* is located on chromosome 13. It consists of 27 exons (with exons 10 and 11 constituting 60 % of the coding region) spanning a region of approximately 70 kb and encodes a protein of 3,418 amino acids.

BRCA1 and BRCA2 are involved in the cellular response to DNA damage intervening both in DNA repair and in the transcriptional regulation of other genes involved in DNA repair and cell-cycle checkpoints activation, thereby preventing the duplication of cells bearing damaged DNA [9–12].

A thorough understanding of the mechanisms by which BRCA1 and BRCA2 maintain genome integrity is crucial to identify non-invasive treatment strategies for women with suspected family predisposition to HBOC [13].

Recently, inhibitors of poly (ADP-ribose) polymerase (PARP), involved in another DNA repair pathway, such as base excision repair (BER), were found to have high efficacy against tumors bearing *BRCA1* or *BRCA2* mutations [14, 15]. Therefore, alterations in *BRCA* genes represent potential biomarkers predictive of the response to chemotherapy of hereditary cancer.

Several criteria have been proposed for the identification of patients who have a hereditary breast and/or ovarian disease, but generally tumors are attributable to this class when there is a family history, onset of disease at a young age, and more than one family member affected.

At present, epidemiologists and geneticists rely on statistical models, among which the most commons are BOADICEA, BRCAPRO, and IBIS. These programs, in the context of genetic counseling, are based on the collection of family medical history information, which allows to calculate the probability of the presence of *BRCA1* and *BRCA2* mutations, whereas genetic testing is performed only if the calculated probability exceeds a predetermined value. In the 90s the implementation of molecular tests for *BRCA1* and *BRCA2* has led to the recognition of a large number of mutations occurring in both genes. Many of these are silent, however, most mutations are small insertions or deletions resulting in non-sense or frame-shift alterations, which lead to a premature termination of translation and, consequently, in a truncated protein. Often the type of mutation is specific to the family/ethnic group (founder effect) [16]. It is possible to find rearrangements of large genomic portions, affecting *BRCA* genes, which lead to altered protein structure. In addition to these mutations there are other frequent amino acid substitutions in BRCA1 and BRCA2, which are defined as “unclassified variants” (UVs) because it is not known whether they can affect gene function and be of considerable clinical significance [17]. Overall, the Breast cancer information core (BIC) database (research.nhgri.nih.gov/bic/) has recorded 1,639 and 1,853 distinct mutations, polymorphisms, and variants in the *BRCA1* and *BRCA2* genes, respectively (data 2010).

Genetic tests have different purposes depending on if they are performed on patients or on their relatives. The complexity that characterizes genomic *BRCA1* and *BRCA2*, their large size, the absence of mutational hot spots, the presence of UVs, and the different distribution of mutations according to geographic areas/ethnic groups, makes the molecular analysis particularly difficult.

There are two different categories of genetic tests: direct and indirect. The study of the complete coding region by direct sequencing is arduous because of the large size of both genes. For this reason, in the past, several indirect techniques have been used as a pre-sequencing screening to identify gene or protein alterations, such as: Protein truncation test (PTT), denaturing high performance liquid chromatography (DHPLC) [18], and High-resolution melting (HRM) analysis [19].

However, the techniques used as indirect tests present several disadvantages, mainly: (a) they are only partially informative; (b) they add further costs to the cost of sequencing, which is in any case necessary for the characterization of the mutation; and (c) they can result in false negatives. The direct sequencing is the only way to characterize specific gDNA alterations although the capillary system based on Sanger methods is still very expensive in terms of time, cost, and knowhow required.

Many efforts are ongoing to improve the performance of the direct method making it faster and cheaper. Here, to this purpose, we designed a new strategy and developed a new cost and time-effective approach for the assessment of *BRCA* mutational status.

## Materials and Methods

### DNA Extraction

Peripheral blood from healthy donors and HBOC patients, recruited at the National Cancer Institute of Naples, was collected, by Vacutainer system, in two 5 ml tubes. The gDNA was extracted in duplicate from EDTA blood samples through the QIAamp DNA maxi kit (Qiagen) according to the supplier’s recommendations.

### DNA Amplification Plate Setup

PCR was performed in 50 μl final volumes, starting from 200 ng of gDNA. The amplification mixture included 1 ×  PCR buffer (Roche), 1 pmol/μl of both forward and reverse primer, 200 μmol of dNTPs, and 2 U of *Taq* DNA polymerase (Roche).

All amplicons were amplified using the following thermal profile. A first denaturation step at 95 °C for 10 min, followed by 40 cycles: denaturation at 94 °C for 30 s, annealing at 60 °C for 30 s, and extension at 72 °C for 1 min. Final extension was accomplished at 72 °C for 10 min.

The entire *BRCA1* and *BRCA2* coding regions were amplified through PCR as previously described [19] with some changes (Table 1). All primer sets, for both genes, were pre-spotted using a Beckman Coulter robotic station BFX liquid handler and dried in 96-well PCR plates through the Eppendorf Concentrator 5301.

**Table 1:** New primer pairs amplifying various *BRCA1*/*2* regions

*BRCA* region	Forward primer sequence	Reverse primer sequence
Ex 1a BRCA1	CCATCCTCTGATTGTACCTTGATT	AGCTTCGGAAATCCACTCTC
Ex 1b BRCA1	AGCTGACAGATGGGTATTCTTTGA	GCACCTCTTCTTCCACAAGGT
Ex 2 BRCA1	AATGAAGTTGTCATTTTATAAACCTTTT	GACATGTCTTTTCTTCCCTAGTATGT
Ex 9 BRCA1	GCTTAACTAGCATTGTACCTGC	CCAAGTCGTGTGTTTACCTATAT
Ex 16 BRCA1	ATTGAAAGTTGCAGAATCTG	TCTTAGTCATTAGGGAGATAC
Ex 10 BRCA2	GAAGGGGTGACTGACCGA	GGTATCTACAACTGTTTCATATAC

*Ex* exon

Negative controls, for all reactions, were pooled in 23 wells.

### PCR Analysis, Products Purification, and Fragment Cloning

All PCR products were analyzed simultaneously through gel electrophoresis (1 % agarose gel in 1 × TBE), run along with a mass and molecular weight marker (Fermentas Mass Ruler 100 bp ladder) to confirm successful amplifications and to check negative controls. The amplicon length ranged between 190 and 903 bp.

The PCR products were then purified using the Millipore HTS multiscreen PCR cleanup plates in automated procedures on a Beckman Coulter BFX liquid handler.

In case of ambiguous results the PCR product was cloned. We re-amplified the specific region using native primers (without M13 tail) and cloned the purified PCR fragments through the Stratagene “StrataClone PCR cloning kit” according to the supplier’s instruction. DNA was extracted from bacterial cell cultures through Sigma “GenElute HP plasmid miniprep kit” according to the supplier’s instructions. The obtained DNA was analyzed by direct sequencing.

### DNA Sequencing and Purification

An automated procedure was developed on a Beckman Coulter BFX liquid handler to setup the sequencing plates (forward and reverse), according to the Applied Biosystem “Big dye Terminator v3.1 Cycle Sequencing kit” manual, and to purify sequence reactions that were subsequently analyzed by capillary electrophoresis on the Applied Biosystems 3730xl DNA Analyzer. To simplify the sequencing reaction we used an appropriate oligo design strategy. All forward primers were designed and synthesized adding, upstream to the sequence complementary to the BRCA region to amplify, the universal M13 forward primer sequence and the same strategy was used for the reverse primer adding the universal reverse M13 primer sequence to the specific BRCA complementary region (M13 forward: CGTTGTAAAACGACGGCCATG and M13 reverse: TTTCACAGGAAACAGCTATGAC).

### Data Analysis

Data analysis was performed through the Applied Biosystems “Variant Reporter™ Software, Version 1.1”, a software that compares the sequence chromatograms with the wild-type sequence, which allowed us to obtain clear results and to standardize the analysis providing general criteria to evaluate and validate the screening.

*BRCA1* (MIM113705) and *BRCA2* (MIM 600185) nomenclature within this article is used as in the BIC database according to GenBank recommendations.

## Results and Discussion

Until now, *BRCA1/BRCA2* mutation analysis has been very difficult, time consuming and expensive, owing to the large size of the two genes and the need to use several various primers, each with a different annealing temperature, for PCR amplification and the subsequent sequencing.

Our analysis was based on the approach of De Leeneer et al. [19] for primer design, although we did not use high-resolution melting analysis (HRM) but direct sequencing. Amplicons were partially different and reduced to 73 (compared to 112).

We developed a PCR-based approach using 73 primers pairs to amplify the complete coding region of *BRCA1* and *BRCA2*; splitting *BRCA1* in 33 merging amplicons, 10 of which encompassing exon 11 and *BRCA2* in 40 merging amplicons, 14 of which encompassing exon 11. This allowed us to use a single 96-wells PCR plate for each sample: *ONE INDIVIDUAL = ONE PLATE PCR*; reducing costs, reducing time, and eliminating a source of potential sample cross contamination due to the concurrent use of more samples.

Moreover, to simplify the procedure, primers were pre-spotted and dried in a 96-well PCR plates immediately available upon analysis request.

Furthermore, all the amplifications were optimized to the same thermal profile. This condition allowed us to carry out all the amplifications in a single experiment.

We performed, at the same conditions used for the 73 sample amplifications, multiplex no-template PCR controls for all PCR experiments. Multiplex controls were prepared by mixing primer sets amplifying products of different lengths in order to easily identify, according to the size, possible contaminating bands, through the agarose gel electrophoretic run. Each mix contained no more than four primer pairs. Each primer was used at the same concentration used for the sample amplification and at the same annealing temperature. Although the efficiency of a multiplex PCR could be different from the efficiency of the single PCR and potentially underestimate a contamination this strategy, using multiplex for negative controls, allowed us to perform in a single PCR plate the analysis of both entire BRCA genes of each patient.

To setup this methodology and for the following testing, we used a pool of gDNAs from 5 different healthy donors. Whereas, to validate the reliability of our procedure, we used 10 gDNAs samples from individuals who were clinically affected by HBOC, whose BRCA mutational status had been formerly characterized by another independent group. We analyzed each of these samples testing all the 73 amplicons corresponding to the entire BRCA1 and BRCA2 coding regions. Our analysis was able to detect and confirm, in a blind way, all the previously identified mutations in both genes.

**Fig. 1:**
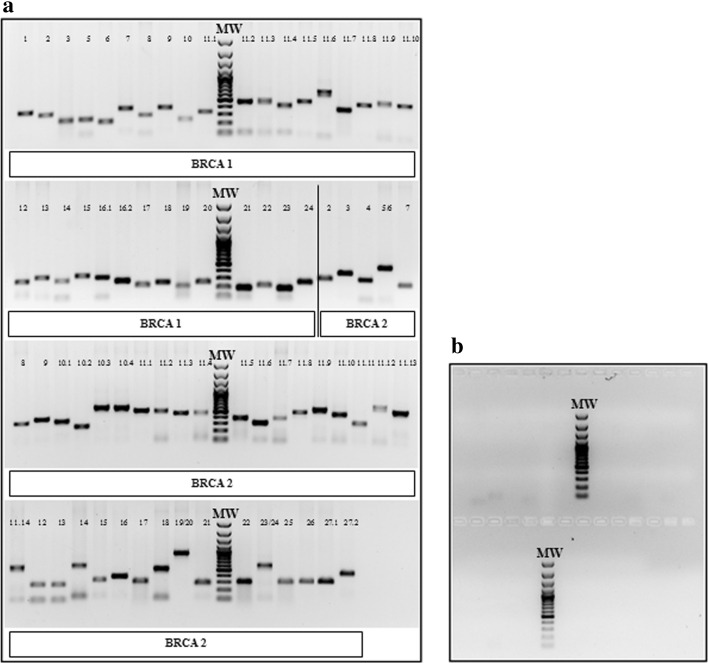
PCR analysis of BRCA1/2 coding regions. **a** 1 % agarose gel electrophoresis of the PCR products to verify the correct amplification of the BRCA1/2 coding regions. MW: molecular weight marker. Each lane corresponds to the amplicon relative to the indicated *BRCA1* or *BRCA2* exon. In particular, for *BRCA1*, we divided exon 11 and exon 16 in ten and two amplicons, respectively (11.1–11.10 and 16.1–16.2); for *BRCA2* we divided exon 10, exon 11, and exon 27 in three, fourteen, and two amplicons, respectively (10.1–10.3; 11.1–11.14, and 27.1–27.2). For BRCA2 only, exon 5 and exon 6, exon 19 and exon 20, exon 23 and exon 24 were amplified within the same amplicon because they were sufficiently short. **b** 1 % agarose gel electrophoresis to control the specificity of amplification. No bands were detected in the mix of negative controls. MW: molecular weight marker

We obtained for all the 15 samples analyzed 73 bands each one of the expected size and without contaminations (Fig. 1a, b).

Once all the amplicons covering the entire coding regions of *BRCA1* and *BRCA2* were obtained, we proceeded to the DNA sequencing phase. All PCR primers were designed to contain at the 5′ end the M13 forward and the reverse sequence to easily perform the sequencing of both strands of the amplicons. This allowed us to simplify the entire sequencing process. For sequence analysis, we compared several Softwares: Lasergene DNA Star; Geneious, CLC, etc. but we found that the Applied Biosystems “Variant Reporter ™Software Version 1.1” was the most handy to analyze mutations. Moreover, this Software provides detailed reports that are very helpful to prepare the response and keep the diagnostic results.

To validate the results, all detected mutations were confirmed with a targeted PCR amplification on a new DNA sample aliquot. In fact, for each patients we performed two DNA extractions separately in two different tubes; then, one aliquot was used to perform the first test while the other one, in the presence of a mutation, was used to confirm the data.

The sequence analysis showed that our methodology of investigation was able to detect and to confirm the 100 % of the mutations previously found in both *BRCA1* and *BRCA2* genes for each of the samples.

We identified mutations in exons 8, 18, and 20 of *BRCA1* and in exon 11 of *BRCA2*.

Insertions or deletions in heterozygosity resulted in the presence of a double sequence. In these cases, to confirm the results, we cloned the amplicon of interest and subsequently we obtained the sequence of each strand. The analysis showed that one strand was wild type whereas the other one bore the mutation.

Analyzing 6 further unknown samples, we found several differences compared to the WT sequences, but only two of these mutations, as resulted from the BIC database, were correlated with the disease. The other identified alterations were either silent, and, therefore, did not modify the protein structure, or reported as of unknown significance by the BIC database. To validate these results we repeated the test, focusing only on the regions containing the two mutations that are known to be associated to the pathology, using the other DNA aliquot as mentioned above.

For all the ten control samples, we never observed a preferential amplification of the wild-type allele; in fact, we were able to identify, in blind, all the mutations previously detected. The direct sequencing method (optimized through the use of the M13 primers and performed with quality controls) was able to correctly identify 100 % of the mutations. Moreover, in the case of unclear sequence results, we proceeded with the amplicon cloning and subsequent sequencing, in order to obtain clear and univocal results on the selected difficult fragment in both alleles. Practically it is not feasible to obtain positive controls for each amplicon; however, the technological approach used implicitly foresees analytical steps. Sequences results are analyzed taking in account single basecalling score for each nucleotide. Moreover, there are no reasons to hypothesize a different mutation detection accuracy in different amplicon, it could be, instead, different in term of performances to detect different mutations like indels or base substitutions. In our control panel samples we tested, with positive results, the ability of our procedure to detect all different types of mutation in homo and hetero zigosity.

Our experimental conditions, which included DNA extraction, PCR amplification and testing through gel electrophoresis, amplicon purification, sequencing reactions, purification of sequencing reaction products, and sequencing by capillary electrophoresis along with overall data analysis, required about one week and a cost of about 1,200 € (corresponding to ~1,500$) including personnel cost and depreciation, maintenance and support of equipment. The experimental conditions refer to the maximum time needed to conduct the investigation of a patient and include the initial analysis (about two days), the validation of data and the possible repetition of the mutated region (about two days), and/or the cloning technique to resolve an ambiguous situations (about three days). The situation is different if the individual subjected to the investigation has a family history with a previously typed mutation. In this case it will be sufficient to study only the region of interest, which will take approximately 1–2 days.

The low cost and the high speed of execution of this method, when compared to the techniques used so far, would allow to perform a more extensive screening, including a greater number of patients, guaranteeing an earlier identification of the risk and the implementation of ad hoc clinical surveillance programs.

The clinical management of women, who are often young, carriers of breast and/or ovarian cancers vary depending on the presence or absence of *BRCA* mutations, which makes negligible the cost of the test when compared to the cost of the therapies. In fact, the screening for *BRCA* mutations could, along with other clinical and pathological information, allow to formulate a personalized therapy in the treatment of breast and/or ovarian cancer [20].

**Fig. 2:**
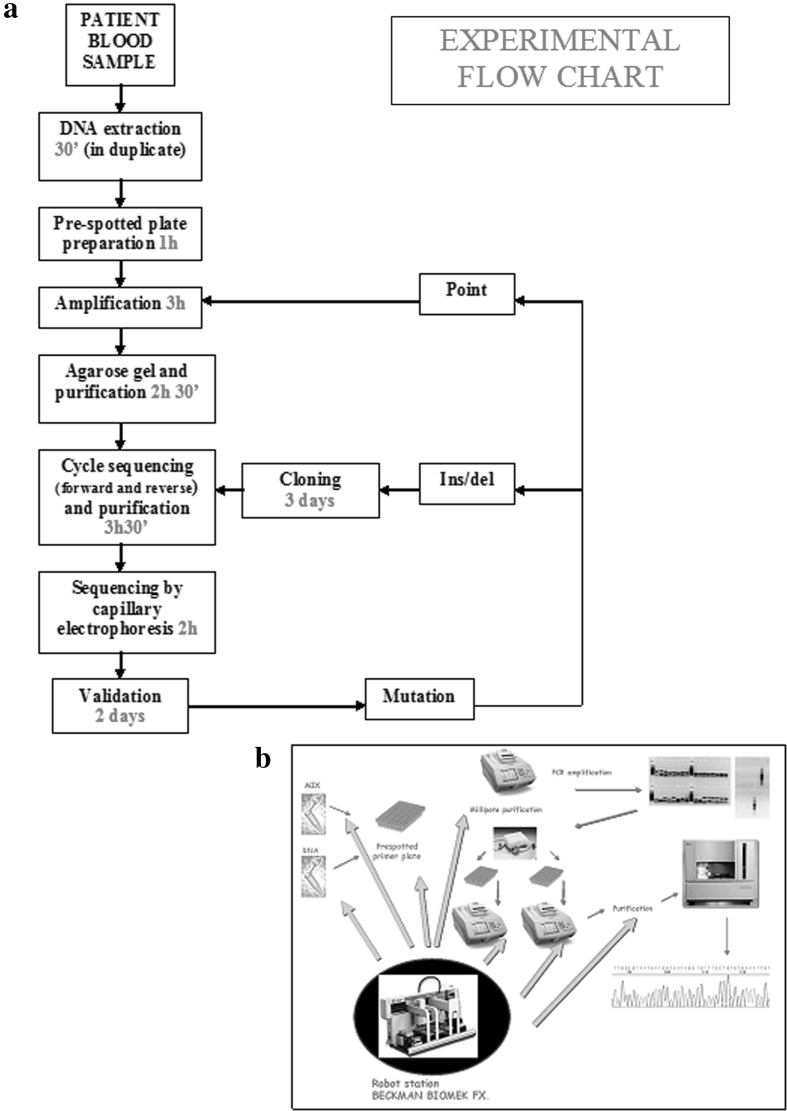
Flow chart showing our experimental conditions. **a** Flow chart showing the timing of all the protocol steps including: DNA extraction, amplification of 73 amplicons covering all the coding regions of *BRCA1* and *BRCA2*, PCR analysis through gel electrophoresis, purification of amplification products, setup of sequencing reaction, purification of sequencing products, and sequencing by capillary electrophoresis. The whole testing requires a time of about 2 working days, representing a rapid and cost-effective method. **b** Schematic representation of the experimental method for *BRCA1/2* testing, showing the key role of the robotic station

In conclusion, we presented a fast and reliable strategy to detect mutations in the *BRCA* genes; this method is very rapid and less expensive than other methods and makes feasible to assess *BRCA* mutational status in the diagnostic setting requiring only ~2 working days (excluding the analysis of the electropherograms) (Fig. 2a, b). This strategy could be readily implemented in a diagnostic laboratory.

In the future, third-generation sequencing technologies, such as those used by PGM ion torrent of Life technologies [21] or Illumina [22], might contribute to lowering the costs of BRCA screening even more, but at present their use for diagnostic purposes is still very controversial and needs further testing.
